# MicroRNA-218-5p regulates inflammation response via targeting TLR4 in atherosclerosis

**DOI:** 10.1186/s12872-023-03124-y

**Published:** 2023-03-08

**Authors:** Jiajuan Chen, Zusheng Tang, Zhen Chen, Yunjie Wei, Hui Liang, Xiaoqiao Zhang, Zhen Gao, Hezhong Zhu

**Affiliations:** 1 Department of Cardiology, Taihe Hospital, Hubei University of Medicine, No. 32 Renminnan Road, Shiyan, 442000 Hubei China; 2 Department of General Practitioner, Taihe Hospital, Hubei University of Medicine, No. 32 Renminnan Road, Shiyan, 442000 Hubei China; 3 Department of Geriatrics, Taihe Hospital, Hubei University of Medicine, Shiyan, 442000 China

**Keywords:** Atherosclerosis, MiR-218-5p, TLR4, Inflammation response

## Abstract

**Background:**

To investigate the expression of miR-218-5p in atherosclerosis patients and its effect on ox-LDL induced THP-1-derived macrophage inflammatory response.

**Methods:**

RT-qPCR detected the expression of serum miR-218-5p, and the diagnostic value of miR-218-5p was analyzed by ROC curve. Pearson correlation coefficient was used to evaluate the correlation between miR-218-5p and CIMT and CRP. THP-1 cells were treated with ox-LDL to construct foam cell model. The expression of miR-218-5p was regulated by in vitro transfection technique, and the effects of miR-218-5p on cell viability, apoptosis and inflammation were investigated. Luciferase reporter genes were used to analyze target genes of miR-218-5p in cell models.

**Results:**

The expression of miR-218-5p in the atherosclerosis cohort was significantly reduced, and miR-218-5p showed a good ability to distinguish patients from healthy people. Correlation analysis showed that the level of miR-218-5p was negatively correlated with the levels of CIMT and CRP. Cytological studies showed that the expression of miR-218-5p in macrophages decreased after ox-LDL induction. ox-LDL treatment on macrophages resulted in decreased cell viability, increased cell apoptosis and production of inflammatory cytokines, which contributed to the exacerbation of plaque formation. However, the above situation was reversed after upregulation of miR-218-5p. Bioinformatics analysis showed that TLR4 may be the target gene of miR-218-5p, and this hypothesis was proved by luciferase reporter gene assay.

**Conclusions:**

The expression of miR-218-5p is reduced in atherosclerosis, and it may regulate the inflammatory response of atherosclerotic foam cells by targeting TLR4, suggesting that miR-218-5p may be a promising target for clinical atherosclerosis therapy.

## Background

The incidence of cardiovascular and cerebrovascular diseases is increasing year by year. According to the World Health Organization (WHO), it is estimated that by 2030, 23.6 million people worldwide will die from cardiovascular and cerebrovascular diseases every year [1]. Atherosclerosis is one of the most important causes of death from cardiovascular and cerebrovascular diseases, and its occurrence is believed to be caused by many factors, including genetic and environmental factors. Known risk factors include hypertension, hypercholesterolemia, diabetes, smoking and so on, among which the increase of cholesterol is an important factor in the occurrence of atherosclerosis [2, 3]. It is worth noting that atherosclerosis is the early developmental stage of coronary heart disease (CHD), which is manifested by reduced arterial elasticity, hardening and thickening of the inner wall of the blood vessels, and the formation of atherosclerotic plaques [4, 5]. The emergency caused by plaque rupture and hemorrhage is the main cause of poor prognosis or death [6]. Therefore, elucidating the mechanism of plaque formation may have positive significance for the prevention and treatment of atherosclerosis.

With the development of gene expression regulation, gene epigenetics has become a new strategy for the prevention and treatment of atherosclerosis. MicroRNAs (miRNAs) are a class of non-coding small molecule single-stranded RNAs with a length of about 18–24 bases [7]. In the evolutionary process, miRNAs are relatively conserved and function mainly by participating in the negative regulation of post-transcriptional gene expression [8]. A large number of studies have shown that miRNA affects the occurrence and development of atherosclerosis in many ways. For example, miR-34a and miR-146a are involved in the progression of atherosclerosis by regulating endothelial cell proliferation and differentiation [9]. miR-126 accelerated the generation of foam cells by affecting the expression of adhesion molecule VCAM-1 [10]. By regulating the differentiation of vascular smooth muscle cells, miR-143 and miR-145 inhibit the dysregulation of smooth muscle cells, thus controlling the progression of atherosclerosis [11]. This study focused on miR-218-5p. MiR-218-5p was originally found to be a tumor suppressor gene whose expression was reduced in a variety of tumors. For example, miR-218-5p impairs the migration and fusion ability of prostate cancer cells by targeting TRAF1. In recent years, researchers have found abnormal expression of miR-218-5p in cardiovascular disease. Ke et al. reported that miR-218-5p was enriched in exosomes derived from endothelial progenitor cells, and exosomes containing a large amount of miR-218-5p could significantly improve the degree of myocardial damage and promote the recovery of myocardial tissue integrity in rats with myocardial infarction [12]. Amanda et al. reported that miR-218-5p showed good clinical diagnostic value in patients with diabetes-induced atherosclerosis [13]. So far, the studies on the regulatory mechanism of miR-218-5p on atherosclerosis are still relatively few.

In this study, we investigated the expression trend of miR-218-5p in patients with atherosclerosis. Subsequently, human mononuclear leukemia cells (THP-1) exposed to ox-LDL were used to prepare an atherosclerosis foam cell model to observe the influence of miR-218-5p on the cell model, and further analyze the specific mechanism of miR-218-5p regulation of foam cells, providing new ideas and experimental support for the treatment of atherosclerosis.

## Methods

### Study population and sample collection

A total of 156 volunteers were recruited for this study, of which 80 were patients with atherosclerosis and 76 were healthy people matching the age and sex of the atherosclerosis group. The inclusion criteria for the atherosclerosis group included: patients diagnosed with carotid atherosclerosis by carotid artery ultrasonography. Exclusion criteria included rheumatic heart disease, recent traumatic surgical history, liver and kidney insufficiency, tumor, and hemorrhagic disease. After all volunteers were enrolled in the group, 5 mL of fasting venous blood was collected in anticoagulation tube, and the serum was separated after centrifugation and stored in −80 °C refrigerator for later use.

This research protocol has been approved by the Ethics Committee of Taihe Hospital, Hubei University of Medicine and follows the guidelines of the Declaration of Helsinki on human experiments. All volunteers have signed written informed consent.

### Cell culture and cell transfection

The human monocyte cell line THP-1 cells were purchased from ATCC. THP-1 cells were incubated in RPMI-1640 medium containing 10 mmol/L HEPES, 10% FBS and 1% penicillin/streptomycin antibiotic mixture, and then incubated for 24 h at 37 °C in a constant temperature incubator containing 5% CO_2_. Subsequently, in order to induce THP-1 cells to become adherent macrophages, the cells were seeded into 6-well plates, and the culture medium involving PMA (phorbol 12-myristate 13-acetate) at a final concentration of 100 ng/mL was added, and the cells was incubated for another 24 h. For cell model construction, the cells were cultured in serum-free medium containing different concentrations of oxidized low-density lipoprotein (ox-LDL) for 24 h based on the previously published methods [14].

For cell transfection, according to the instructions of the kit, miR-218-5p mimic and mimic-negative control (mimic-NC) were transfected into THP-1 cells using Lipofectamine 2000 transfection reagent. The transfection efficiency was detected by RT-qPCR 6 h after transfection.

### RT-qPCR

Total RNA was extracted from THP-1 cells using TRIzol. Next, reverse transcription was performed using the TransScript® One-Step RT-PCR kit and TransScript® MiRNA First-Strand cDNA Synthesis kit, strictly following the kit instructions. Then, PCR reaction was performed to amplify the gene fragment. Reaction conditions are as follows: 95 °C of pre-denaturation for 20 s, 1 cycle, followed by 40 cycles of denaturing for 10 s at 95 °C, annealing for 20 s at 60 °C and extending for 10 s at 70 °C. U6 was used as internal reference, and the relative expression of genes was calculated by 2^−ΔΔCt^ method.

### Cell apoptosis

Annexin V-FITC (fluorescein isothiocyanate)/PI (propidium iodide) Apoptosis detection kit is used to detect apoptosis. Simply put, 48 h after transfection, cells were collected with trypsin and cleaned with PBS for 2–3 times. After the cells were re-suspended with the binding buffer, 10 muL FITC and 5 muL PI solutions were added to the cell suspension and incubated at room temperature for 15 min away from light. Finally, cell apoptosis was detected by flow cytometry at excitation wavelength of 488 nm and emission wavelength of 630 nm.

### Cell viability

Cell viability was evaluated by CCK-8 assay. Cells were inoculated into 96-well plate at a density of 2 × 10^4^ cells/well. After transfection and ox-LDL induction, 10 nM CCK-8 solution was added to the cells at predetermined time points (0 h, 24 h, 48 h, 72 h). After incubation in the dark for 1 h, the OD value at 450 nm was detected by microplate reader.

### Enzyme-linked immunosorbent assay

ELISA was used to perform cytokine concentration determination. The cell supernatant was collected and the levels of interleukin-1β (IL-1β) (catalogue number: CSB-E08053h), IL-6 (catalogue number: CSB-E04638h), and tumor necrosis factor-α (TNF-α) (catalogue number: CSB-E04740h) were measured using the commercially available ELISA kits (CUSABio, Wuhan, China) according to the manufacturer's instructions.

### Luciferase reporter gene

THP-1 cells were inoculated into 24-well plates for cell culture. In brief, a wild-type TLR4-3’UTR fragment containing the putative binding region of miR-218-5p or a mutated TLR4-3’UTR fragment containing the putative binding region of miR-218-5p was inserted into the luciferase reporter gene plasmid. Then, the above reporter plasmids and miR-218-5p mimic or miR-218-5p inhibitor were co-transfected into cells. After 48 h of transfection, luciferase activity was measured using the luciferase reporting analysis system (Promega, Madison, WI, USA) according to the manufacturer's instructions. The luciferase activities were normalized to Renilla fluorescence.

### Data analysis

SPSS 22.0 software was used for data analysis in this study. According to the normality of the data (using Kolmogorov–Smirnov test), parametric t-test or non-parametric Mann–Whitney U test was performed. Receiver operating characteristic (ROC) curve was used to evaluate the clinical diagnostic accuracy of abnormally expressed miR-218-5p in atherosclerosis. The value of Pearson correlation coefficient analysis is to evaluate the correlation between the level of miR-218-5p and the index. Data are presented as mean ± standard deviation [15]. *P* < 0.05 was defined as significantly different.

## Results

### Comparison of general information and clinical features

Table 1 summarized the basic information and clinical characteristics of healthy population and atherosclerosis population. The results showed that there was no significant difference in gender, age, body mass index (BMI), high-density lipoprotein cholesterol (HDL-C), fasting blood-glucose (FBG), systolic blood pressure (SBP) and diastolic blood pressure (DBP) between the two groups (*P* > 0.05). Additionally, the levels of total cholesterol (TC), triglyceride [16], low density lipoprotein cholesterol (LDL-C), carotid intima-media thickness (CIMT) and C-reactive protein (CRP) in the atherosclerosis group were significantly higher than those in the control group (*P* < 0.001).

**Table 1:** Basic clinical information of the subjects

Indicators	Control (n = 76)	Atherosclerosis (n = 80)	*P* value
Gender (males/females)	54/22	55/25	0.876
Age (years)	60.29 ± 9.69	61.16 ± 11.59	0.928
BMI (kg m^−2^)	25.23 ± 2.57	25.39 ± 2.62	0.674
TC (mmol/L)	3.53 ± 0.73	5.71 ± 0.66	< 0.001
TG (mmol/L)	1.51 ± 0.27	2.27 ± 0.33	< 0.001
LDL-C (mmol/L)	2.31 ± 0.39	2.61 ± 0.43	< 0.001
HDL-C (mmol/L)	1.91 ± 0.42	1.69 ± 0.37	0.195
FBG (mmol/L)	5.45 ± 0.32	5.48 ± 0.31	0.917
SBP (mm Hg)	121.70 ± 15.61	123.87 ± 16.94	0.691
DBP (mm Hg)	82.70 ± 5.96	83.17 ± 5.79	0.457
CIMT (mm)	0.51 ± 0.14	1.23 ± 0.18	< 0.001
CRP (mg/L)	6.45 ± 1.67	10.36 ± 1.85	< 0.001

*BMI* body mass index, *TC* total cholesterol, *TG* triglyceride, *HDL-C* high-density lipoprotein cholesterol, *LDL-C* low density lipoprotein cholesterol, *FBG* fasting blood-glucose, *SBP* systolic blood pressure, *DBP* diastolic blood pressure, *CIMT* carotid intima-media thickness, *CRP* C-reactive protein. Data are expressed as n or mean ± standard deviation (SD)

### Expression of miR-218-5p and its diagnostic value in atherosclerosis

**Fig. 1:**
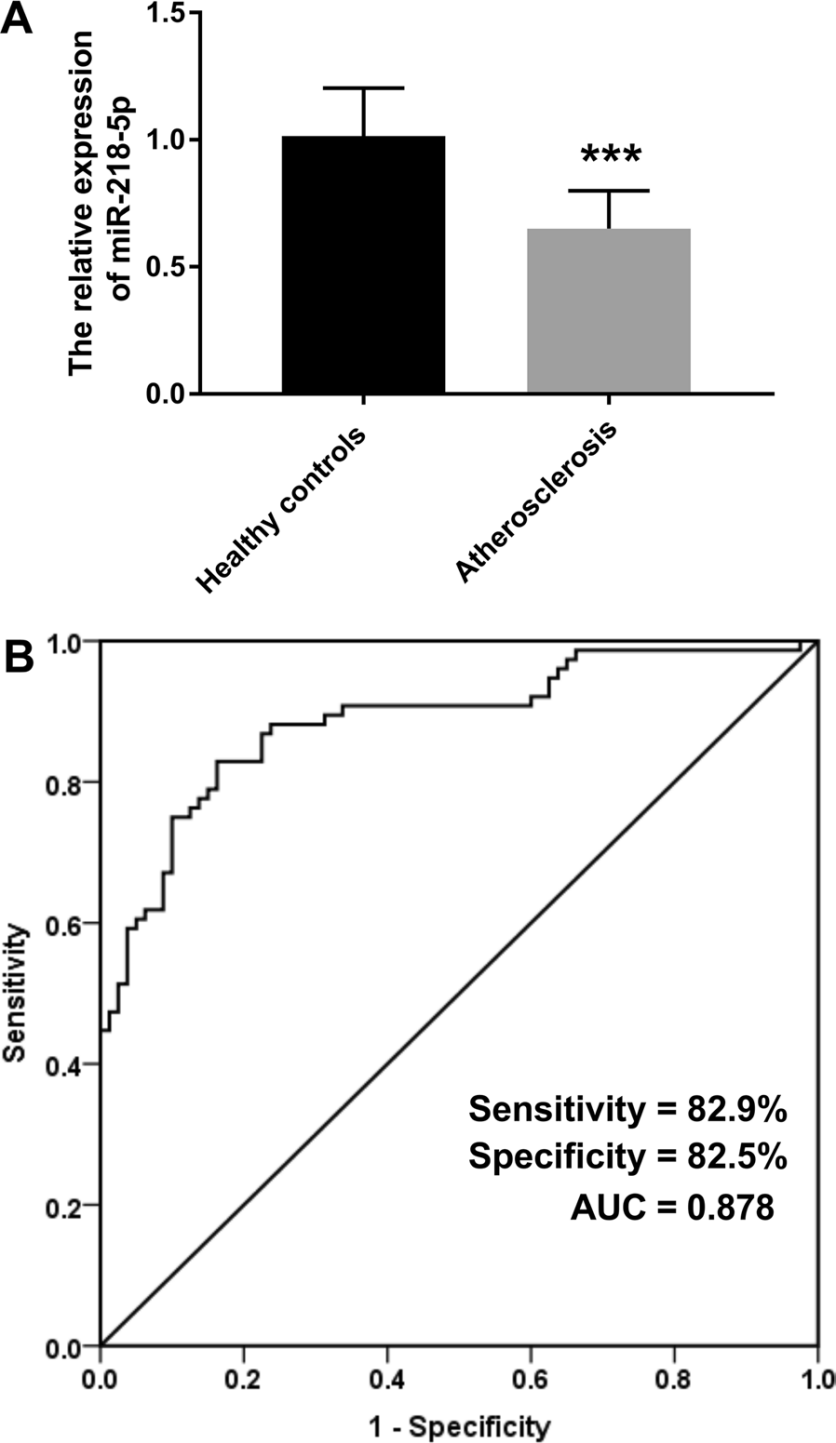
Expression of miR-218-5p and its diagnostic value in atherosclerosis. **(A)**. The expression of miR-2180-5p in serum of atherosclerosis patients was reduced compared with controls. **(B)**. The abnormal expression of miR-218-5p shows high clinical diagnostic value for atherosclerosis. ^***^P < 0.001

Detection of miR-218-5p in serum of all subjects was performed by PCR. It was observed from the results that the expression of miR-218-5p in the atherosclerosis cohort was significantly reduced compared with the control group, preliminarily indicating that the abnormality of miR-218-5p may be associated with the occurrence of atherosclerosis (Fig. 1A, *P* < 0.001). Additionally, ROC curve was constructed to evaluate the clinical diagnostic value of miR-218-5p in atherosclerosis. As shown in Fig. 1B, it can be intuitively seen that the area under the curve (AUC value) of miR-218-5p is 0.878, and the sensitivity and specificity are 82.9% and 82.5%, respectively, indicating that the reduced expression of miR-218-5p has a high accuracy in clinical diagnosis of atherosclerosis.

### Correlation of miR-218-5p with CIMT and CRP

**Fig. 2:**
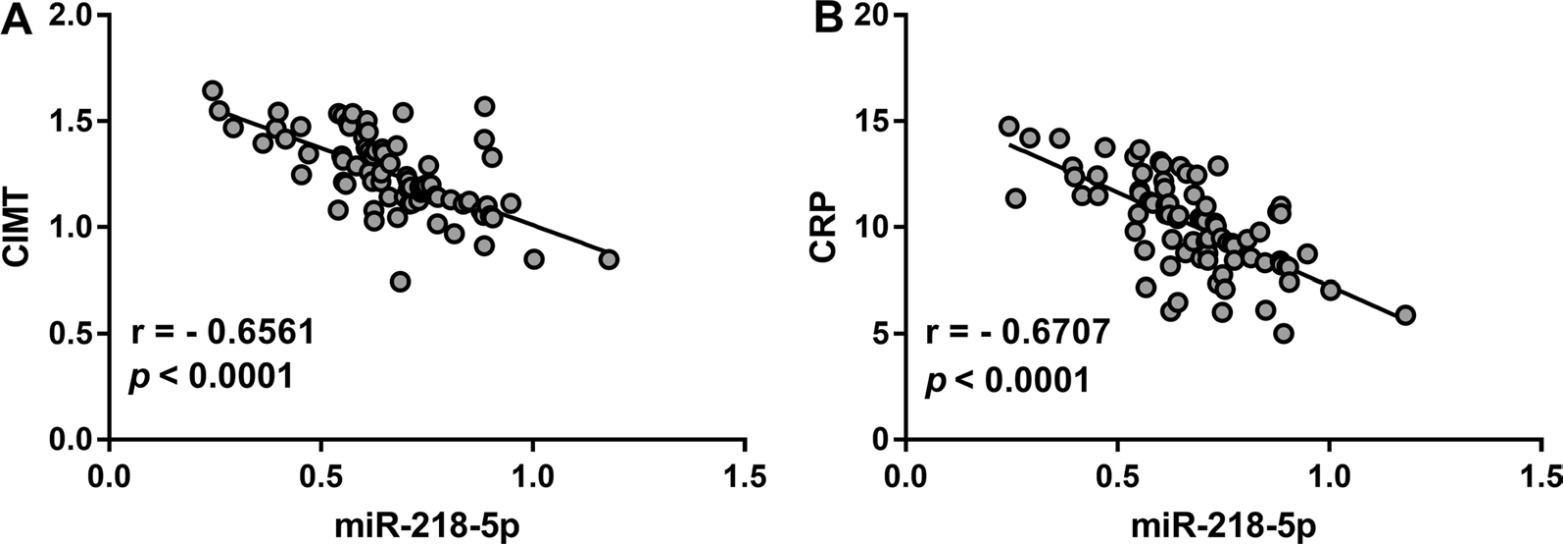
Correlation of miR-218-5p with CIMT and CRP. The level of miR-218-5p was significantly negatively correlated with **(A)**. CIMT and **(B)**. CRP values in patients with atherosclerosis

The correlation of miR-218-5p with CIMT and CRP was evaluated by Pearson correlation coefficient analysis. As exhibited in Fig. 2A–B, the expression level of miR-218-5p was negatively correlated with the levels of CIMT (r = −0.6561, *P* < 0.001) and CRP (r =−0.6707, *P* < 0.001).

### Effects of miR-218-5p on macrophage viability and apoptosis

**Fig. 3:**
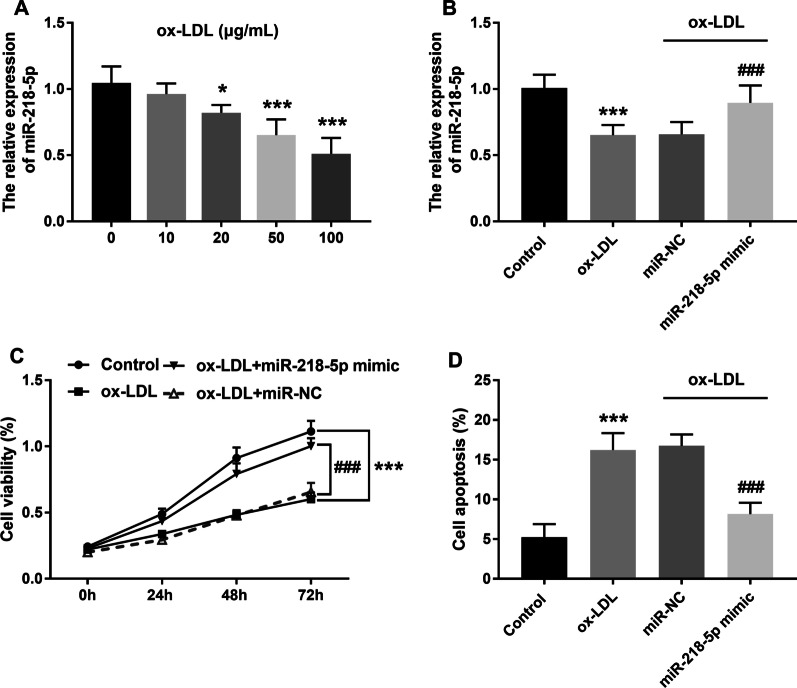
Effects of miR-218-5p on macrophage viability and apoptosis. **(A)**. Expression of miR-218-5p in macrophages induced by ox-LDL at different concentrations. **(B)**. ox-LDL induction of macrophages resulted in reduced miR-218-5p expression, while transfection with miR-mimic increased miR-218-5p level. **(C)**. Upregulated expression of miR-218-5p could significantly improve cell viability. **(D)**. Overexpression of miR-218-5p attenuated ox-LDL-induced apoptosis. ^*^P < 0.05, ^***^P < 0.001 vs. Control group. ^###^*P* < 0.001 vs. ox-LDL group

The THP-1 macrophage inflammation model was constructed by ox-LDL induction. Compared with the 0 μg/mL group (blank control group), the expression of miR-218-5p in the 20, 50 and 100 μg/mL groups was significantly reduced after ox-LDL stimulation at different concentrations. In order to minimize the toxic and side effects of ox-LDL on cells and simulate the reaction of macrophages to ox-LDL under physiological conditions, 50 μg/mL was selected as the optimal stimulation concentration on the premise of causing sufficient inflammatory response (Fig. 3A, *P* < 0.001). After receiving ox-LDL stimulation, the expression of miR-218-5p in macrophages was significantly decreased. However, when transfected with miR-218-5p mimics and then treated with ox-LDL, the content of miR-218-5p in macrophages increased (Fig. 3B, *P* < 0.001). For cell function, ox-LDL treatment showed a strong inhibitory effect on cell viability and promoting effect on cell apoptosis. However, after the up-regulated expression of miR-218-5p, the adverse effect of ox-LDL on cells was significantly weakened, which was manifested as the enhancement of cell viability and inhibition of apoptosis (Fig. 3C–D, *P* < 0.001).

### Effects of miR-218-5p on the inflammatory response of macrophages

**Fig. 4:**
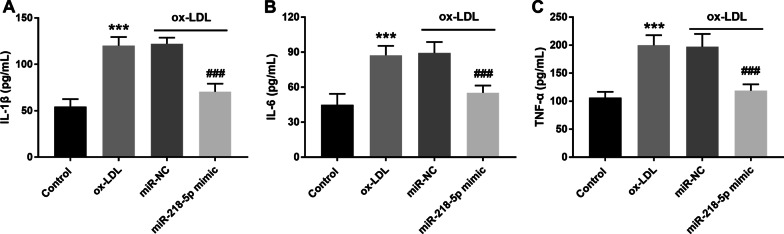
Effects of miR-218-5p on the inflammatory response of macrophages. Upregulating the level of miR-218-5p can inhibit the promotion of ox-LDL on the inflammatory response of macrophages and reduce the production of cytokines, including **(A)**. IL-1β, **(B)**. IL-6, and **(c)**. TNF-α. ^***^P < 0.001 vs. Control group. ^###^*P* < 0.001 vs. ox-LDL group

ELISA is used to detect the inflammatory response of macrophages. The results showed that ox-LDL treatment induced the inflammatory response of macrophages, and the production of IL-1β, IL-6 and TNF-α increased. When the deletion of miR-218-5p was offset by the appearance of miR-218-5p mimic, the degree of inflammatory reaction was decreased, which was mainly manifested in minished cytokine production (Fig. 4A–C, *P* < 0.001).

### Prediction and verification of miR-218-5p target genes

**Fig. 5:**
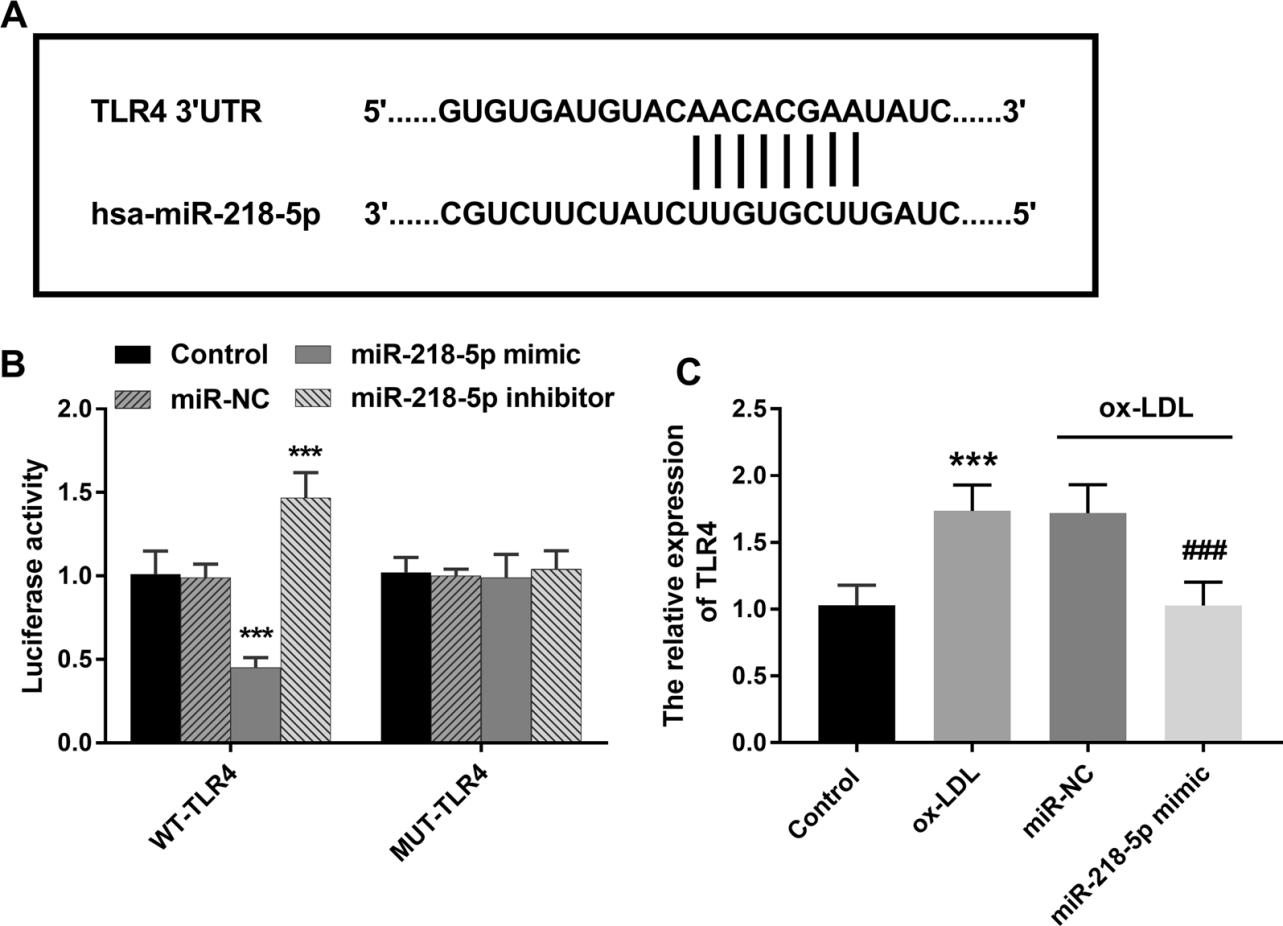
Prediction and verification of miR-218-5p target genes. **(A)**. Complementary binding sites of miR-218-5p and TLR4. **(B)**. Luciferase reporter gene assay confirmed that miR-218-5p directly targets TLR4. **(C)**. TLR4 expression was increased in ox-LDL-induced THP-1 cells. ^***^P < 0.001 vs. Control group

The online database TargetSacn predicted that there were complementary sites between miR-218-5p and TRL4, and we speculated that TRL4 was its downstream target gene. The sequences of both miR-218-5p and TRL4 were shown in Fig. 5A. The results of luciferase reporter gene assay showed that transfection of miR-218-5p mimic or inhibitor could weaken or enhance the luciferase activity of WT-TLR4 group (Fig. 5B, *P* < 0.001), but it had no effect on the luciferase activity of MUT-TLR4 group. In addition, in the cell model, the stimulation of ox-LDL induced the increased expression of TLR4, while the overexpression of miR-218-5p significantly down-regulated the expression of TLR4, which further verified the targeting relationship between miR-218-5p and TLR4 (Fig. 5C, *P* < 0.001).

## Discussion

More and more basic and clinical studies have confirmed that miRNA plays a key role in atherosclerotic diseases. They may be involved in various links of atherosclerosis by regulating inflammatory signaling pathways, affecting the synthesis and secretion of inflammatory factors. In this study, we evaluated the expression and clinical value of miR-218-5p in patients with carotid atherosclerosis. The expression of miR-218-5p was reduced in the atherosclerosis group, and this abnormal expression pattern showed an advantage in distinguishing atherosclerosis from healthy individuals. Besides, this study showed that in the lipid indexes of clinical subjects, the levels of TC, TG and LDL-C in the atherosclerosis group were significantly higher than those in the healthy control group, which further proved that high blood lipid content was associated with the occurrence and development of atherosclerosis. Studies have shown that elevated TG may promote atherosclerosis by influencing the expression of LDL. In fact, TG in blood mainly exists in TG-rich lipoproteins [17]. When the level of TG increases, the activity of cholesterol ester transfer protein increases, and the TG in lipoprotein is hydrolyzed by hepatic triglyceride lipase, forming a small and dense LDL deposit on the blood vessel wall, which is the driving force to promote the progress of atherosclerosis [18].

Macrophage-derived foam cells are the most abundant immune cells in atherosclerotic plaques [19]. Macrophage-derived foam cells play a key regulatory role in plate formation and development during the pathophysiological process of atherosclerosis. Atherosclerotic plaque develops from the lipid-striation stage. After endothelial cells are activated, monocytes in blood migrate into the subcutaneous space through adhesion molecules and chemokines recruitment, and then differentiate into macrophages [16]. LDL is considered to be a key initiating factor in the development of atherosclerosis. After oxidative modification, LDL becomes ox-LDL, and the ox-LDL recognition receptor on the surface of macrophages mediates the phagocytosis and uptake of ox-LDL by macrophages to form foam cells [20]. After apoptosis or death of the foam cells, a large amount of lipids was deposited on the blood vessel wall to form atherosclerotic plaques [21]. The proliferation and apoptosis of foam cells have a profound impact on the occurrence and development of vascular inflammation and the evolution and prognosis of atherosclerosis.

In this study, the atherosclerotic cell model was constructed by exposing THP-1 macrophages to ox-LDL, and the reduced expression of miR-218-5p in the cell model was subsequently detected by PCR, which was consistent with the trend in clinical studies. With further research, we evaluated the effect of miR-218-5p on the atherosclerotic cell model. After ox-LDL treatment, we found that the activity of macrophages was significantly inhibited, and the number of apoptotic cells was significantly increased, meanwhile, ox-LDL treatment promoted the inflammatory response of macrophages. Studies have shown that with the development of atherosclerosis, the efferocytosis is gradually overloaded, and the apoptosis of macrophage-derived foam cells promotes the formation of plaque necrosis core, increases the instability of plaque, and promotes the occurrence of cardiovascular inflammation [22]. Furthermore, based on the theoretical model of atherosclerosis, immune inflammation is another key mechanism driving the development of atherosclerosis. All cells that contribute to atherogenesis, including macrophages, white blood cells, and smooth muscle cells, produce inflammatory cytokines that promote plaque growth, such as IL-1β, TNF-α, and IL-6 [15]. Here, we subsequently found that upregulating the expression of miR-218-5p had positive significance in improving cell viability, inhibiting cell apoptosis and production of inflammatory factors. According to these results, we speculated that miR-218-5p can prevent the formation of plaques and maintain the stability of plaques by regulating inflammatory response and inhibiting apoptosis. Obviously, animal experiments should be further considered and improved for the particularity of macrophage apoptosis.

Studies on the regulatory mechanism of miR-218-5p on some cardiovascular diseases have been gradually carried out. MiR-218-5p has been reported to have a protective effect on vascular endothelial cells by inhibiting apoptosis of endothelial cells in Henoch Purpura via targeting HMGB1 [23]. MiR-218-5p has been confirmed to have a good diagnostic value for the occurrence and recurrence of coronary heart disease [24]. In addition, miR-218-5p has been demonstrated to have an inhibitory effect on the proliferation of cardiac fibroblasts in myocardial fibrosis [25]. In this study, we learned that Toll-like receptor 4 (TLR4) is the downstream target gene of miR-218-5p through bioinformatics analysis and luciferase reporter gene, and the two molecules are mutually targeted. This result is consistent with the study of Cui et al., who found that miR-128-5p promoted the occurrence of inflammation by up-regulating TLR4 expression in PC12 cells treated with high glucose [26]. The TLR4 gene, located on the long arm of human chromosome 9 (9q33.1), is a family of receptors that mediate inflammation [27]. Studies have reported that TLR4 binds to ligands to form dimers to initiate downstream inflammatory reactions. In addition, after the synthesis of TLR4 by immune cells, it can activate nuclear factor κB (NF-κB) through signal transduction pathway, leading to the production of a large number of inflammatory factors [28]. Yang et al. reported elevated levels of TLR4 and NF-κB in atherosclerotic rabbit models fed a high fat diet [29]. This study preliminarily indicated that miR-218-5p in macrophage-derived foam cells may affect cell function and inflammatory response through up-regulation of TLR4 expression, and then participate in the development of atherosclerosis. The specific role of TLR4 in macrophage-derived foam cells still needs to be further verified.

There are two limitations in this study that need to be clarified. One is that although the modeling of THP-1 induced by ox-LDL is well known, the possible molecular mechanism by which ox-LDL regulates miR-218-5p in THP-1 remains unclear. It is necessary to further study this field to better clarify the relationship between miR-218-5p and ox-LDL. The levels of receptors associated with LDL, such as PCSK9, which are important genes involved in cholesterol metabolism, can be investigated in follow-up experiments. The other is that the role of TLR4 has not been further studied. The increase of TLR4 in cell models is speculated to have an effect on ox-LDL-induced THP-1 cells, and subsequent experiments should be conducted to improve this result.

## Conclusions

In conclusion, the expression of miR-218-5p was decreased in both atherosclerosis patients and atherosclerosis cell models. In vitro studies have shown that miR-218-5p regulates apoptosis and inflammation of macrophage-derived foam cells by targeting TLR4, thus promoting the progression of atherosclerosis. This study enriched the research on the mechanism of atherosclerosis and provided data support for the prevention and treatment of atherosclerosis at the level of gene epigenetics.

## Data Availability

The datasets used and/or analysed during the current study are available from the corresponding author (Hezhong Zhu: zhuhezhong08@163.com; Zhen Gao: zgunu3wo@163.com) on reasonable request.

## Abbreviations

BMI: Body mass index
CHD: Coronary heart disease
CIMT: Carotid intima-media thickness
CRP: C-reactive protein
DBP: Diastolic blood pressure
FBG: Fasting blood-glucose
HDL-C: High-density lipoprotein cholesterol
IL-1β: Interleukin-1β
LDL-C: Low density lipoprotein cholesterol
mimic-NC: Mimic-negative control
miRNAs: MicroRNAs
NF-κB: Nuclear factor κB
ox-LDL: Oxidized low-density lipoprotein
ROC: Receiver operating characteristic
SBP: Systolic blood pressure
TC: Total cholesterol
TNF-α: Tumor necrosis factor-α
WHO: World Health Organization
